# The Moderating Role of Race/Ethnicity in Suicide Risk and Family Connectedness in Youth Presenting to the Emergency Department

**DOI:** 10.1016/j.jaacop.2024.10.009

**Published:** 2025-01-31

**Authors:** Ritika Merai, Tesia Shi, August X. Wei, Donna A. Ruch, Jeffrey A. Bridge, Maryland Pao, Lisa M. Horowitz

**Affiliations:** a Intramural Research Program at the National Institute of Mental Health, National Institutes of Health, Bethesda, Maryland; b Ohio State University College of Medicine and Abigail Wexner Research Institute at Nationwide Children’s Hospital, Columbus, Ohio

**Keywords:** ED, family connectedness, race/ethnicity, suicide prevention, youth

## Abstract

**Objective:**

Significant racial disparities exist in youth suicide rates. Research has identified family connectedness as a strong protective factor against suicide. However, the role of family in youth mental health can vary based on cultural factors that may differ across race and/or ethnicity. This study aimed to evaluate how race/ethnicity moderates the association between suicide risk and family connectedness.

**Method:**

This secondary analysis of Emergency Department Screen for Teens at Risk for Suicide (ED-STARS) study 1 included youth ages 12 to 17 years. Data were obtained for race/ethnicity, family connectedness (combined score of 2 items, range 2 [low] to 10 [high]), and the Ask Suicide-Screening Questions (ASQ) tool. Binary logistic regression assessed the association between family connectedness and positive ASQ screen, with race/ethnicity as a moderator.

**Results:**

Data for 5,514 participants (50.9% female, 45.8% non-Hispanic White, mean [SD] age = 15.0 [1.7] years) were analyzed. Of all participants, 23.5% (1,293/5,514) screened positive for suicide risk. Overall, participants reported high family connectedness (mean [SD] = 8.2 [1.74]). Multiracial participants had the lowest average family connectedness (7.93) and the highest screen positive rate (28.34% [70/247]). For a 1-unit increase in family connectedness, the odds of screening positive were significantly lower for Black/African American participants (odds ratio 0.54, 95% CI 0.49-0.59) compared to White participants (odds ratio 0.46, 95% CI 0.43-0.49) (difference: *z* = −3.17, *p* = .001).

**Conclusion:**

The protective effect of family connectedness for suicide risk may vary by race/ethnicity. In this study, family connectedness was less protective against suicide risk for Black/African American youth compared to White youth. Findings highlight the importance of cultural considerations in family-based interventions for suicide prevention.

## Plain language summary

There are significant racial disparities in youth suicide rates, with family connectedness as one of the strongest protective factors against suicide. This study evaluated how race/ethnicity moderates the relation between suicide risk and family connectedness using data from the Emergency Department Screen for Teens at Risk for Suicide (ED-STARS) study which included youth aged 12-17 years. Family connectedness may be less protective against suicide risk for Black/African American youth compared to White youth. In addition, multiracial youth were at higher risk with the lowest average family connectedness and the highest screen positive rate for suicide risk. These results highlight the importance of cultural considerations in family-based interventions for suicide prevention.

There are significant racial disparities in suicide rates in youth ages 12 to 17 years in the United States. American Indian and Alaska Native (AI/AN) youth have the highest rates of suicide, and the rates for Black youth are rapidly increasing compared to other races.^1^ Additionally, youth and young adults have higher rates of emergency department (ED) visits for self-harm compared with middle-aged adults,^2^ with the largest increase in psychiatric ED utilization found for Black and Latinx youth.^3^ Historically, most research about suicidal thoughts and behaviors has focused on White populations due to lower suicide rates reported among people of color. However, these trends are now changing, with increasing rates of suicidal thoughts and behaviors in other racial and/or ethnic groups. Currently, existing suicide research does not adequately address prevention, identification, or interventions for these vulnerable populations.^4^

Several risk and protective factors have been identified for suicidality among youth.^5^ Risk factors such as discrimination, stigma associated with help-seeking and mental illness, and lack of access to health care may especially affect people with minoritized identities, including youth of color. Conversely, protective factors include effective coping; a strong sense of cultural identity; reduced access to lethal means; cultural, religious, or moral objections to suicide; and feeling connected to others.

Family connectedness has been established as one of the strongest protective factors against suicide risk. Family support has been shown to significantly reduce the association between intersecting marginalized identities and suicidality, framing a potential target for intervention.^6^ The term connectedness has been operationalized in various ways in research and is often interchangeably used with terms attachment, support, and bonding.^7^ Based on the Family Connectedness Scale,^8^^,^^9^ an adolescent’s sense of family connectedness refers to their perception of interpersonal relations with their family. This can include feelings of closeness, respect, and understanding and being able to confide in one’s family. Family connectedness is associated with lower levels of emotional and behavioral problems. Youth who felt more connected to parents reported lower levels of depressive symptoms and suicidal ideation.^10^ Another study with at-risk adolescents found that the relation between self-esteem and suicide risk among adolescents is influenced by their level of family support. Specifically, when adolescents have low family support, their level of self-esteem plays a more significant role in mitigating suicide risk compared to when they have high family support.^11^

Although family connectedness has been shown to have broad protective effects for youth, cultural and racial factors may impact its protective effect on mental health specifically for families of color. For example, one study reported that adolescents from European backgrounds tend to emphasize independence and autonomy while still valuing familial relationships, whereas adolescents from Chinese backgrounds tend to show connectedness through respect for authority, obedience, and fulfilling family obligations.^12^ Another study examining suicidal behaviors among Hispanic girls discussed the unique convergence of cultural and familial factors that arise from the conflict between traditional family obligations and Western individualistic culture.^13^ Due to these cultural differences in family values, there may be differences in how families discuss and view mental health. A study including Black youth found that adolescents with parental support were more likely to report suicidal ideation, but not suicide attempts.^14^ This suggests that these adolescents may perceive more risk associated with disclosing suicide attempts. Additionally, many cultural groups manifest and interpret distress differently and may not verbalize suicidal thoughts or intent as readily as other groups.^15^ Whereas family connectedness is generally high for AI/AN,^16^ Hispanic/Latino,^17^ Asian,^18^ and Black^14^^,^^19^ communities due to the high emphasis on family-centered relationships in these cultures, they also have high levels of stigma^20^^,^^21^ and low help-seeking behaviors.^17^^,^^21^ Thus, despite having strong family relationships, family connectedness may not be as protective against suicide risk for youth of color.

Understanding how risk and protective factors intersect for communities of color could better inform culturally responsive suicide prevention interventions. The current study has 2 aims: (1) to describe suicide risk and family connectedness within various racial/ethnic groups; and (2) to evaluate how race/ethnicity moderates the association between suicide risk and family connectedness. We hypothesize that because mental health is particularly stigmatized in families of color, the association between suicide risk and family connectedness will vary by race/ethnicity, with family connectedness being less protective against suicide risk for youth of color (who identified as non-White) compared to White youth.

## Method

### Study Sample

This study is a secondary analysis using data from the first cohort of the Emergency Department Screen for Teens at Risk for Suicide (ED-STARS) study, which developed an algorithm for the Computerized Adaptive Screen for Suicidal Youth (CASSY).^22^ Data were collected from patients ages 12 to 17 who were recruited between June 2015 and July 2016 at 13 pediatric EDs in the Pediatric Emergency Care Applied Research Network (PECARN) across the United States. Each participant completed self-reported surveys with 92 primary questions and up to 27 additional questions at baseline. More detailed information on the ED-STARS study can be found elsewhere.^22^ Participants who completed the primary measures of interest were included in this study.

### Measures

#### Demographics

Age, sex (male/female), and racial and/or ethnic identity were obtained by participant self-report or parent/guardian report. The categories for race/ethnicity were as follows: Hispanic/Latino, non-Hispanic (NH) AI/AN, NH Asian American, NH Native Hawaiian and Pacific Islander, NH Black/African American (Black/AA), NH multiracial (selected more than one race), and NH White. Asian American and Native Hawaiian and Pacific Islander (AANHPI) participants were combined into one group due to small sample sizes.

#### Suicide Risk: Ask Suicide-Screening Questions

Suicide risk was assessed by the first 4 questions of the Ask-Suicide Screening Questions (ASQ) tool.^23^ The first 4 questions of the ASQ measure recent and past suicidal ideation and behavior, with a yes or no binary response to each question. The ASQ has robust psychometric properties, with a sensitivity of 96.9% and specificity of 87.6% in the original study. Participants who screened positive (a “yes” response to any of the questions) and who screened negative (a “no” response to all of the questions) were included in the study.

#### Family Connectedness

Family connectedness was measured using 2 items adapted from the Family Connectedness Scale.^8^^,^^9^ The 2 adapted items were “How much do people in your family understand you?” and “How much does your family pay attention to you?” Likert scale responses ranged from 1 (not at all) to 5 (very much). Mean family connectedness scores were derived for each question. Based on prior research,^24^ a combined score was derived by the sum of the 2 questions (range 2 [low] to 10 [high]) for analysis.

### Statistical Analyses

Binary logistic regression assessed the association between family connectedness and ASQ positive screens, with race/ethnicity as a moderator, controlling for age and sex. NH White participants were used as the reference group in the moderation analysis. Additional exploratory analysis was conducted to compare each racial/ethnic group with one another. Odds ratios (ORs) with 95% CIs were calculated for each race/ethnicity. All analyses were conducted using R Studio version 4.2.2 (R Foundation for Statistical Computing, Vienna, Austria).

## Results

**Figure 1:**
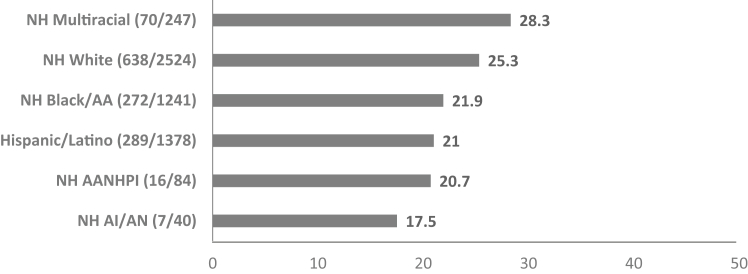
Screen Positive Rate for Suicide Risk Within Each Racial/Ethnic Group ***Note:****AA = African American; AANHPI = Asian American Native and Hawaiian Pacific Islander; AI/AN = American Indian/Alaska Native; NH = non-Hispanic.*

Data were analyzed from 5,514 (50.9% female, 45.8% NH White, mean [SD] age = 15.0 [1.7] years) of 6,536 participants from the original study (Table 1). Of the participants, 23.5% (1,293/5,514) screened positive for suicide risk. Figure 1 shows the screen positive rates for each racial/ethnic group. Participants reported high levels of family connectedness as measured by 2 adapted items, with a mean (SD) of 8.2 (1.74) out of 10. AI/AN and AANHPI youth had the highest mean family connectedness scores (8.53 and 8.46 out of 10, respectively). Overall, participants had lower scores for family connectedness on question 1 (“How much do people in your family understand you?”; mean score = 3.95) than on question 2 (“How much does your family pay attention to you?”; mean score = 4.30). Mean family connectedness scores for both questions as well as the combined scores for each racial/ethnic group are displayed in Table 2. Across groups, multiracial youth had the lowest average family connectedness (7.93 out of 10) and the highest screen positive rate for suicide risk (28.3% [70/247]).

**Table 1:** Demographic Information (5,514 Participants)

Variables	Value	
	Mean	(SD)
Age, y (range 12-17 y)		
	14.96	(1.65)
	**n**	**(%)**
Sex		
Male	2,061	(37.4)
Female	2,811	(51)
Missing data	642	(11.6)
Race/ethnicity		
Hispanic/Latino	1,378	(25)
NH AANHPI	84	(1.5)
NH AI/AN	40	(0.7)
NH Black/AA	1,241	(22.5)
NH multiracial	247	(4.5)
NH White	2,524	(45.8)
Chief complaint		
Medical complaint	3,694	(67)
Unintentional injury	1,032	(18.7)
Psychiatric complaint	783	(14.2)
Unknown	5	(0.1)

Note: AA = African American; AANHPI = Asian American and Native Hawaiian Pacific Islander; AI/AN = American Indian/Alaska Native; NH = non-Hispanic.

**Table 2:** Mean Family Connectedness Scores for Each Racial/Ethnic Group

Race/ethnicity	Family Connectedness					
	Q1: “How much do people in your family understand you?” (range 1-5)		Q2: “How much does your family pay attention to you?” (range 1-5)		Combined score for Q1 and Q2 (range 2-10)	
	Mean	(SD)	Mean	(SD)	Mean	(SD)
NH multiracial	3.75	(1.13)	4.19	(0.90)	7.93	(1.84)
NH Black/AA	3.89	(1.10)	4.27	(0.92)	8.16	(1.82)
NH White	3.91	(1.06)	4.27	(0.83)	8.18	(1.70)
Hispanic/Latino	3.95	(1.08)	4.34	(0.86)	8.29	(1.73)
NH AANHPI	4.08	(0.96)	4.38	(0.77)	8.46	(1.56)
NH AI/AN	4.17	(0.96)	4.35	(0.77)	8.52	(1.63)

Note: AA = African American; AANHPI = Asian American and Native Hawaiian Pacific Islander; AI/AN = American Indian/Alaska Native; NH = non-Hispanic; Q1 = question 1; Q2 = question 2.

**Figure 2:**
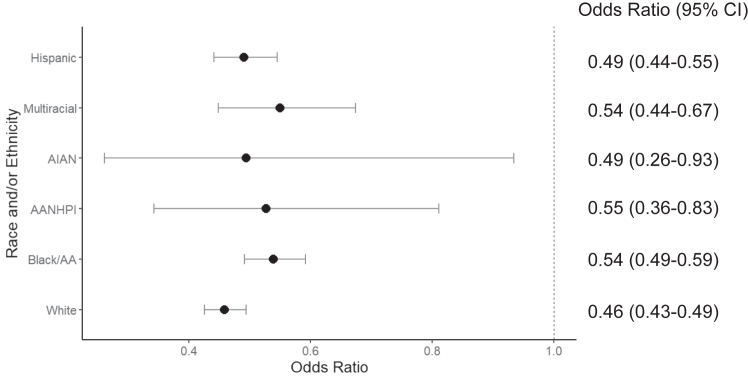
Odds Ratios for Each Racial/Ethnic Group for a Positive Suicide Risk Screen With a 1-Unit Increase in Family Connectedness ***Note:****Odds ratio <1 indicates a decrease in the odds. AA = African American; AANHPI = Asian American Native and Hawaiian Pacific Islander; AI/AN = American Indian/Alaska Native.*

An increase in family connectedness decreased the odds of a positive suicide risk screen for all racial/ethnic groups. The association between family connectedness and suicide risk differed between Black/AA youth and White youth (Table 3). For every 1-unit increase in family connectedness, the odds of a positive suicide risk screen decreased by 54% for White youth (OR 0.46, 95% CI 0.43-0.49) and 46% for Black/AA youth (OR 0.54, 95% CI 0.49-0.59; difference: *z* = −3.17, *p* = .001). Odds ratios for all racial/ethnic groups are displayed in Figure 2.

**Table 3:** Binary Logistic Regression Model With a Positive Suicide Risk Screen as the Outcome, Family Connectedness as the Predictor, and Race/Ethnicity as a Moderator, Controlling for Age and Sex

	*b*	SE	*z*	*p*
FC	−.78	0.04	−20.55	∗∗∗
Race/ethnicity				
NH White	Reference	Reference	Reference	Reference
NH Black	−1.48	0.47	−3.17	∗∗
NH AANHPI	−1.50	1.73	−0.87	.38
NH AI/AN	−.99	2.55	−1.39	.70
NH multiracial	−1.24	0.86	−1.44	.15
Hispanic/Latino	−.75	0.51	−1.49	.14
Interactions				
FC × NH White	Reference	Reference	Reference	Reference
FC × NH Black	.16	0.06	2.67	∗∗
FC × NH AANHPI	.18	0.22	0.85	.39
FC × NH AI/AN	.08	0.33	0.23	.82
FC × NH multiracial	.17	0.11	1.25	.13
FC × Hispanic/Latino	.07	0.07	1.02	.31
Sex				
Male	Reference	Reference	Reference	Reference
Female	.82	0.09	9.56	∗∗
Age	.04	0.02	1.42	.16

Note: AA = African American; AI/AN = American Indian/Alaska Native; AANHPI = Asian American Native and Hawaiian Pacific Islander; FC = Family Connectedness; NH = non-Hispanic.

∗ *p* < .05; ∗∗*p* < .01; ∗∗∗*p* < .001.

When comparing each racial/ethnic group, there was a detectable difference between White and Black/AA youth. White youth had lower odds of screening positive for suicide risk compared with Black/AA youth (OR 0.85, 95% CI 0.96-0.75). For all other comparisons, CIs included 1, indicating no detectable differences between the groups. Odds ratios for all pairwise comparisons are in Figure S1, available online.

## Discussion

This exploratory secondary analysis examined the association between suicide risk and family connectedness across race/ethnicity in a sample of youth presenting to the ED. Family connectedness had a protective effect for suicide risk across the sample, where an increase in family connectedness was associated with decreased odds of screening positive for suicide risk. This finding is consistent with previous research supporting family connectedness as a protective factor for suicide. However, an increase in family connectedness was found to be less protective against suicide risk for Black/AA youth compared to White youth. For other racial/ethnic groups, the protective effects of family connectedness did not differ when compared with White youth. Of all groups, family connectedness appeared to be least protective for AANHPI and multiracial participants. Multiracial youth had the highest screen positive rate and lowest family connectedness scores. Conversely, AANHPI and AI/AN youth reported the highest family connectedness scores and lowest screen positive rates for suicide risk.

The lower protective effects of family connectedness for Black youth in this sample warrants further research on other factors that may impact family dynamics in this group. Previous research has shown that suicide rates have increased for Black youth, additional risk factors such as systemic racism, trauma, and exposure to violence and death.^25^ Together, these findings highlight that Black/AA youth are at a higher risk for suicide and imply that conventional family-based interventions might not fully address the unique needs of Black/AA youth. One example of a family-based intervention grounded in racial socialization is Engaging, Managing, and Bonding through Race (EMBRace).^26^ This program can be used to address race-based stress and trauma among Black youth and families as part of clinical care and can be evaluated for further effects on suicide prevention.^27^

Similarly, more research is needed on targeted family interventions for multiracial youth, who had the highest screen positive rate for suicide risk in this study. Research has shown that multiracial youth have worse mental health outcomes compared with their monoracial peers with higher levels of anxiety, depressive symptoms,^28^ and suicidal thoughts and behaviors.^29^ Multiracial youth may also have multiple minoritized racial identities, making them more vulnerable to external racial and cultural factors impacting suicide risk. Given that the multiracial population is highly heterogeneous, future research should examine how mental health outcomes can vary based on subgroups and contexts within this population.^29^

Among other racial/ethnic groups, AI/AN, AANHPI, and Hispanic/Latino youth had the highest average family connectedness, which is consistent with prior research regarding strong, family-centered social relationships and networks in these communities.^16^, ^17^, ^18^ Although AI/AN youth in this study had the lowest screen positive rates, they maintain the highest national suicide rates among all racial/ethnic groups. The low screen positive rates found in this study may be due to the low sample size. It may also be partly explained by lack of disclosure of suicide risk due to medical mistrust^30^ and the taboo surrounding discussions about death in some AI/AN communities.^31^

Overall, a quarter of the sample screened positive for suicide risk, which is higher than rates typically seen in the ED.^32^ This may be due to selection bias in enrollment given that ED-STARS was a study on suicide risk. Notably, 14% of the sample presented with psychiatric chief complaints, including suicidal ideation, which may have contributed to higher positive screen rates.

The findings of this study are preliminary and should be interpreted with consideration of the following limitations. First, low sample sizes for most racial/ethnic groups limited the ability to detect a difference between White participants and participants from most other racial/ethnic groups. The sample sizes for AI/AN and AANHPI groups were the lowest, limiting the ability to make strong conclusions about their screen positive rates and family connectedness. Racial/ethnic groups were also divided into heterogeneous categories such as multiracial, AANHPI, and Hispanic/Latino, limiting the generalizability of the findings. Additionally, no measures were used to assess cultural factors and barriers, limiting the scope of interpreting some findings. Second, family connectedness was measured using only 2 self-reported and adapted items that do not capture the various aspects of family connectedness; thus, a low score on these items may not necessarily indicate a less supportive family environment for youth. Findings on family connectedness in this sample may also be unique to the setting, as primary caretakers of youth had to provide consent for participation in the research study. These factors may influence why participants had high perceived family connectedness during this time, especially in the survey item measuring familial attention. Third, the study design is cross-sectional, preventing establishment of directionality of the relations.

Future studies should assess the association between family connectedness and suicide risk in other settings, such as primary care and schools, with a more racially diverse sample. Future studies should also focus on racial/ethnic data disaggregation to fully capture the heterogeneity present within youth and families from multiracial, AANHPI, and Hispanic communities. Additional research with a more in-depth assessment of family connectedness among different racial/ethnic groups can inform culturally responsive family-based interventions for suicide prevention.

Findings from this study underscore the importance of family connectedness as a protective factor for suicide risk. Although extant research on youth mental health established protective effects of family connectedness, more studies are needed on how race/ethnicity may impact family connectedness and suicide risk, especially among youth of color. In this sample of youth presenting to the ED, family connectedness was less protective for Black youth. These findings highlight the importance of cultural considerations in suicide prevention research, identification, and development of tailored family-based interventions.

## CRediT authorship contribution statement

**Ritika Merai:** Writing – review & editing, Writing – original draft, Visualization, Methodology, Formal analysis, Conceptualization. **Tesia Shi:** Writing – review & editing, Writing – original draft, Formal analysis, Conceptualization. **August X. Wei:** Writing – review & editing, Writing – original draft, Methodology. **Donna A. Ruch:** Writing – review & editing. **Jeffrey A. Bridge:** Writing – review & editing. **Maryland Pao:** Writing – review & editing, Writing – original draft, Supervision. **Lisa M. Horowitz:** Writing – review & editing, Writing – original draft, Supervision, Conceptualization.

## References

[1] Centers for Disease Control and Prevention (2024). WISQARS—Web-based Injury Statistics Query and Reporting System. https://www.cdc.gov/injury/wisqars/index.html.

[2] Centers for Disease Control and Prevention (2024). Health Disparities in Suicide. https://www.cdc.gov/suicide/disparities/?CDC_AAref_Val=https://www.cdc.gov/suicide/facts/disparities-in-suicide.html.

[3] Kalb L.G., Stapp E.K., Ballard E.D., Holingue C., Keefer A., Riley A. (2019). Trends in psychiatric emergency department visits among youth and young adults in the US. Pediatrics.

[4] Oshin L., Hausmann-Stabile C., Meza J.I. (2022). Suicide and suicidal behaviors among minoritized youth. Child Adolesc Psychiatr Clin N Am.

[5] Centers for Disease Control and Prevention (2024). Risk and Protective Factors for Suicide Prevention. https://www.cdc.gov/suicide/risk-factors/index.html.

[6] Standley C.J., Foster-Fishman P. (2021). Intersectionality, social support, and youth suicidality: a socioecological approach to prevention. Suicide Life Threat Behav.

[7] Whitlock J., Wyman P.A., Moore S.R. (2014). Connectedness and suicide prevention in adolescents: pathways and implications. Suicide Life Threat Behav.

[8] Resnick M.D., Bearman P.S., Blum R.W. (1997). Protecting adolescents from harm. Findings from the National Longitudinal Study on Adolescent Health. JAMA.

[9] Eisenberg M.E., Resnick M.D. (2006). Suicidality among gay, lesbian and bisexual youth: the role of protective factors. J Adolesc Health.

[10] Foster C.E., Horwitz A., Thomas A. (2017). Connectedness to family, school, peers, and community in socially vulnerable adolescents. Child Youth Serv Rev.

[11] Sharaf A.Y., Thompson E.A., Walsh E. (2009). Protective effects of self-esteem and family support on suicide risk behaviors among at-risk adolescents. J Child Adolesc Psychiatr Nurs.

[12] Hardway C., Fuligni A.J. (2006). Dimensions of family connectedness among adolescents with Mexican, Chinese, and European backgrounds. Dev Psychol.

[13] Zayas L.H., Pilat A.M. (2008). Suicidal behavior in Latinas: explanatory cultural factors and implications for intervention. Suicide Life Threat Behav.

[14] Boyd D.T., Quinn C.R., Jones K.V., Beer O.W.J. (2022). Suicidal ideations and attempts within the family context: the role of parent support, bonding, and peer experiences with suicidal behaviors. J Racial Ethn Health Disparities.

[15] Goldston D.B., Molock S.D., Whitbeck L.B., Murakami J.L., Zayas L.H., Hall G.C. (2008). Cultural considerations in adolescent suicide prevention and psychosocial treatment. Am Psychol.

[16] Mpofu J.J., Crosby A., Flynn M.A. (2023). Preventing suicidal behavior among American Indian and Alaska Native adolescents and young adults. Public Health Rep.

[17] Villatoro A.P., Morales E.S., Mays V.M. (2014). Family culture in mental health help-seeking and utilization in a nationally representative sample of Latinos in the United States: the NLAAS. Am J Orthopsychiatry.

[18] Lam E.L., Kandula N.R., Shah N.S. (2023). The role of family social networks in cardiovascular health behaviors among Asian Americans, Native Hawaiians, and Pacific Islanders. J Racial Ethn Health Disparities.

[19] Nguyen A.W., Taylor R.J., Chatters L.M., Taylor H.O., Lincoln K.D., Mitchell U.A. (2017). Extended family and friendship support and suicidality among African Americans. Soc Psychiatry Psychiatr Epidemiol.

[20] Goodwill J.R., Zhou S. (2020). Association between perceived public stigma and suicidal behaviors among college students of color in the U.S. J Affect Disord.

[21] Clement S., Schauman O., Graham T. (2015). What is the impact of mental health-related stigma on help-seeking? A systematic review of quantitative and qualitative studies. Psychol Med.

[22] King C.A., Brent D., Grupp-Phelan J. (2021). Prospective development and validation of the computerized adaptive screen for suicidal youth. JAMA Psychiatry.

[23] Horowitz L.M., Bridge J.A., Teach S.J. (2012). Ask Suicide-Screening Questions (ASQ): a brief instrument for the pediatric emergency department. Arch Pediatr Adolesc Med.

[24] Arango A., Brent D., Grupp-Phelan J. (2024). Social connectedness and adolescent suicide risk. J Child Psychol Psychiatry.

[25] Sheftall A.H., Vakil F., Ruch D.A., Boyd R.C., Lindsey M.A., Bridge J.A. (2022). Black youth suicide: investigation of current trends and precipitating circumstances. J Am Acad Child Adolesc Psychiatry.

[26] Anderson R.E., McKenny M.C., Stevenson H.C. (2019). EMBRace: developing a racial socialization intervention to reduce racial stress and enhance racial coping among Black parents and adolescents. Fam Process.

[27] Alvarez K., Polanco-Roman L., Samuel Breslow A., Molock S. (2022). Structural racism and suicide prevention for ethnoracially minoritized youth: a conceptual framework and illustration across systems. Am J Psychiatry.

[28] Fisher S., Reynolds J.L., Hsu W.-W., Barnes J., Tyler K. (2014). Examining multiracial youth in context: ethnic identity development and mental health outcomes. J Youth Adolesc.

[29] Oh H., Winn J.G., Li Verdugo J. (2024). Mental health outcomes of multiracial individuals: a systematic review between the years 2016 and 2022. J Affect Disord.

[30] Guadagnolo B.A., Cina K., Helbig P. (2009). Medical mistrust and less satisfaction with health care among Native Americans presenting for cancer treatment. J Health Care Poor Underserved.

[31] Colclough Y.Y., Brown G.M. (2014). American Indians’ experiences of life-threatening illness and end of life. J Hosp Palliat Nurs.

[32] Roaten K., Horowitz L.M., Bridge J.A. (2021). Universal pediatric suicide risk screening in a health care system: 90,000 patient encounters. J Acad Consult Liaison Psychiatry.

