# Supplementary figures and images for: The Moderating Role of Race/Ethnicity in Suicide Risk and Family Connectedness in Youth Presenting to the Emergency Department
Source: JAACAP Open. 2025 Jan 31;3(3):448–54. doi: 10.1016/j.jaacop.2024.10.009 (PMC12414300; doi:10.1016/j.jaacop.2024.10.009)

## Slide 1
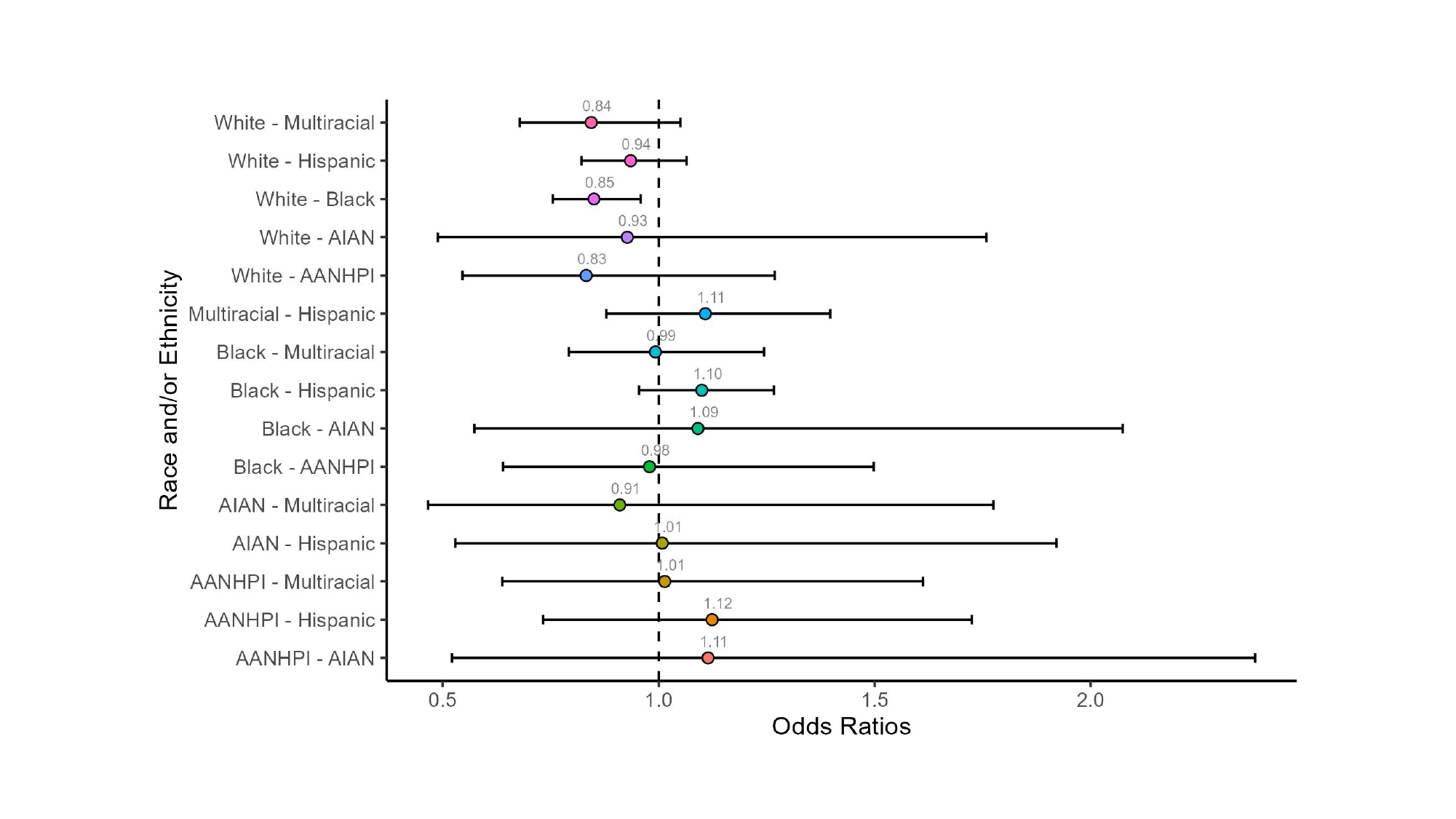

Supplement: Supplementary Figure [file mmc1.pptx]
